# Not all information is equally important: informed consent to genetic testing for hereditary cancer

**DOI:** 10.1007/s00432-026-06422-y

**Published:** 2026-01-27

**Authors:** Paula Thomas, Sven Asmussen, Katharina Klein, Nicolas Straub, Stephanie Stegen, Christoph Kowalski, Stephen Schüürhuis, Dorothee Speiser, Markus A. Feufel, Friederike Kendel

**Affiliations:** 1 Department of Anesthesiology and Intensive Care Medicine, Charité—University Medical Center Berlin, Augustenburger Platz 1, 13353 Berlin, Germany; 2 Faculty of Law, Chair of Civil Law (Prof Dr Gerhard Wagner), Commercial Law and Economics, Humboldt-Universität zu Berlin, Unter den Linden 6, 10099 Berlin, Germany; 3 Hereditary Breast and Ovarian Cancer Center, Charité—University Medical Center Berlin, Charitéplatz 1, 10177 Berlin, Germany; 4 Deutsche Krebsgesellschaft e. V., Kuno-Fischer-Straße 8, 14057 Berlin, Germany; 5 BRCA-Netzwerk e. V., Thomas-Mann-Str. 40, 53111 Bonn, Germany; 6 Institute of Biometry and Clinical Epidemiology, Charité—University Medical Center Berlin, Charitéplatz 1, 10177 Berlin, Germany; 7 Department of Psychology and Ergonomics (IPA), Division of Ergonomics, Technische Universität Berlin, Straße des 17. Juni 135, 10623 Berlin, Germany

**Keywords:** Informed consent, Genetic counseling, Personal genetic information, Data protection, Hereditary diseases, Patient-Centered care

## Abstract

**Purpose:**

Informed consent in medical care is supposed to guarantee patient autonomy. However, in practice, written consent is often inadequate for this purpose: In an effort to meet legal requirements, consent forms are often comprehensive and complex. They cover all information that could potentially be relevant, possibly overwhelming patients rather than addressing their concerns. Thus, there is an urgent need for more patient-centered consent forms. As a first step toward this goal, this study assessed the importance of various aspects of consent to genetic testing from the patients’ perspective.

**Methods:**

A cross-sectional online study was conducted with 224 participants at elevated risk for hereditary breast and/or ovarian cancer. Participants rated the importance of 14 aspects typically covered on the consent form. Each aspect was compared with all other aspects using multiple contrast tests for repeated measures. Participants also provided hypothetical consent to each aspect. Voluntary comments to the consent aspects were analyzed using qualitative content analysis.

**Results:**

Although the majority of consent aspects were rated important in absolute terms, we observed relative differences. Specifically, consent aspects reflecting a clinical benefit for the patient and her family were rated as more important relative to all other aspects. This included, for example, consent to receiving additional test results which could imply further clinical consequences.

**Conclusion:**

Our results may inform the communication between patients and their counseling physicians prior to genetic testing. They also provide an empirical basis for revising consent forms to better align with patients’ needs while remaining legally sound.

**Supplementary Information:**

The online version contains supplementary material available at 10.1007/s00432-026-06422-y.

## Introduction

The ideal of the informed patient creates an ethical dilemma. In theory, the combination of mandatory disclosure and written informed consent (IC) is intended to maximize patient autonomy. In practice, however, the opposite often appears to be the case. In an effort to provide the most comprehensive information possible while ensuring legal protection, patients are confronted with an ever-increasing amount of information.

In medical ethics, respect for autonomy has become paramount: the right of individuals to make decisions about their own bodies. There has been a shift away from the paternalistic model, where physicians make decisions on behalf of their patients, toward patient-centered care, where the individual’s values, preferences, and needs are central to clinical decision-making (Emanuel and Emanuel 1992; Krones and Richter 2008). IC is intended to ensure that decisions reflect the patient’s preferences and wishes. Written consent formalizes the prior verbal explanation (O’Neill 2003). It consolidates all essential information, serving as proof of the informed decision, the protection of the patient’s rights and the legal protection of the healthcare provider (O’Neill 2003).

The active role that IC assigns to patients places considerable demands on them (Berger et al. 2009). Advances in medical procedures have led to increasingly complex treatments, often involving significant risks and a range of possible alternatives. With advancements in genetic diagnostics, the possibilities are becoming ever more extensive, making full understanding all the more crucial to preserve patient autonomy.

For the complexity of written consent, testing for the detection of a genetic mutation for hereditary breast and/or ovarian cancer will serve as a paradigmatic example in this article. Consider a young woman with family members who tested positive for a *BRCA1* mutation. If she inherited this mutation, it may significantly increase her lifetime risk of developing breast and/or ovarian cancer. Thus, before she declares her consent to testing, she must be informed about the available preventive and prophylactic measures, the limitations of testing, psychosocial implications and implications for her future life and that of her family. The way in which the sensitive data are processed and documented is also important. IC to genetic testing thus requires information about a wide range of aspects relating to medical benefits and risks as well as data processing, documentation, and legal compliance. This information is provided during the counseling session, where questions and concerns can be addressed. Topics such as biobanking, the possibility of secondary findings and future use of genetic data further increase the complexity of IC .

### Consent to clinical data use is the default

In various medical settings, patients at the same time grant a (at least) dual consent: one for the medical procedure itself, for instance a blood test and the analysis of the test material, and a separate one for the more technical aspects – particularly the use of their clinical data. The General Data Protection Regulation (GDPR) (EU) 2016/679 provides the central legal framework for data protection in the European Union. Under the GDPR, any use of personal data is generally prohibited unless explicitly permitted by law (Official Journal of the European Union. Legislation. 2016). Such permission may derive from statutory authorizations (Art. 6(1)(b)–(f) GDPR), i.e. existing legal regulations for acts of data processing, or from the specific consent of the data subject (Art. 6(1)(a) GDPR), here: the patient.

In clinical practice, consent is the most commonly used legal basis to justify the use of patient data. This practice reflects a legal preference as well as the broader cultural and ethical assumption that asking the patient for consent is the most transparent and patient-centered approach. Other available legal, i.e. statutory, authorizations are largely neglected (Uecker 2019), despite their potential applicability (Metzger et al. 2023).

In Germany, data privacy and protection in the field of genetic testing is not regulated solely by the GDPR but also by the Genetic Diagnostics Act (*Gendiagnostikgesetz*, GenDG). The GenDG was enacted to prevent misuse and discrimination, and to safeguard the rights of the individuals concerned (Gendiagnostikgesetz (GenDG) – Gesetz Über Genetische Untersuchungen Bei Menschen 2019).

### Limitations of informed consent as a regulatory tool

IC and mandatory disclosure have been the primary regulatory answer to many policy questions in contemporary legislation related to data privacy and protection (Ackermann 2009; Ben-Shahar and Schneider 2014). The ideal underlying this approach is that the best way to protect people’s interests is to provide them with all relevant information and to assume that they can make an informed decision based on it (Ben-Shahar and Schneider 2014).This aligns with legal standards and supports transparency and accountability. However, the objective of providing individuals with exhaustive information (Berger et al. 2009) ultimately appears to lead to cognitive overload, which, in turn, undermines IC (Goldschmitt et al. 2025; Han 2021; Hofmann 2023). Empirical legal studies support this observation, showing that privacy notices are rarely read by users, as they tend to be time-consuming and difficult to understand (Ben-Shahar and Chilton 2016; Sprigman and Tontrup 2024).

### The gap between regulatory intent and practice

In clinical practice, the discrepancy between normative ideals and practical realities carries particular weight due to the direct implications for patient autonomy and professional responsibility. Several factors have been identified as impairing IC. Firstly, patients can be overwhelmed by the sheer volume of information provided (Brand et al. 2019; Chandrasekharan and Taggart 2011) or simply lose interest in the details - a phenomenon referred to as ‘consent fatigue’ (Geller et al. 2022; Ploug and Holm 2016). As a result, when confronted with multiple consent aspects at once, individuals often respond reflexively and without meaningful consideration (Doherty et al. 2017; Geller et al. 2022). Other patients may try to understand the information but struggle with its complexity (Han 2021; Schwaegermann et al. 2021).

Taken together, the sheer volume and complexity of information undermine the very purpose of IC by reducing it to an almost automatic, procedural formality. As Ben-Shahar and Schneider succinctly put it, one of the central reasons for the failure of mandated disclosure regimes is that: ‘Most people find disclosures complex, obscure, and dull’ (Ben-Shahar and Schneider 2014, p. 79). This problem is particularly serious in the medical context. If consent forms no longer provide a clear and reliable basis for informed decisions, IC risks losing its essential purpose of protecting patients’ rights and autonomy (Beauchamp and Childress 2019; Ben-Shahar and Schneider 2014).

Ben-Shahar and Schneider (2014) state that disclosure obligations rarely work in reality and cannot be salvaged in the long term, even through improved design or simplification. They therefore recommend critically examining which problems actually require regulation and developing alternative approaches if necessary (Ben-Shahar and Schneider 2014). We build on this argument and assume that the information provided in consent forms differs in its perceived importance to patients. This assumption is likely to be particularly relevant for issues of data privacy and protection, which constitute the central focus of our study. The findings may inform the development of consent forms that are both patient-centered and legally sound, thereby enhancing the overall quality of IC in genetic medicine and related fields.

## Methods

### Study design

We conducted a cross-sectional online questionnaire study which is reported according to STROBE (Von Elm et al. 2007) and CHERRIES (Eysenbach 2012) guidelines.

### Sample and recruitment

Recruitment of patients at elevated risk for hereditary breast and/or ovarian cancer took place from May to July 2023 und was carried out through BRCA-Netzwerk e.V. (patient support group) and the Center for Hereditary Breast and Ovarian Cancer (HBOC) at Charité – University Medical Center Berlin. All patients had undergone genetic counseling in the past. 89.5% (*n* = 191) had undergone genetic testing. Access to the internet and a PC or mobile device were required to fill out the online questionnaire.

### Data collection and procedure

The questionnaire was distributed via Research Electronic Data Capture^®^ (REDCap^®^) web application. Participants were informed about the aims, duration (15–20 min) and voluntariness of participation. The study was approved by the Ethics Committee at Charité – University Medical Center Berlin (EA4/160/44).

### Materials

We reviewed consent forms by HBOC-centers or human genetic laboratories in Berlin to get an overview of the data privacy and processing aspects included in such consent forms. After categorizing the contents provided across the consent forms, 14 aspects were identified (see Table 1).

**Table 1:** Consent aspects typically asked of patients when deciding for or against genetic testing

Consent aspect	Description
Consent aspect 1	Duration of storage of genetic test result
Consent aspect 2	Use of remaining test material for research purposes
Consent aspect 3	Notification of additional test results
Consent aspect 4	Access to genetic data by employees within a medical practice or center
Consent aspect 5	Disclosure of test result to other treating physicians
Consent aspect 6	Contact by telephone
Consent aspect 7	Contact by e-mail
Consent aspect 8	Contact for participation in medical follow-up studies
Consent aspect 9	Contact for re-evaluation of test result
Consent aspect 10	Digital storage of genetic data
Consent aspect 11	Digital storage of genetic data in general hospital information system (HIS)
Consent aspect 12	Including genetic data in medical records
Consent aspect 13	Transfer of health data to a university in a non-EU country to calculate the individual risk of disease
Consent aspect 14	Use of test result for counseling of biological relatives

During the study, participants were provided with an explanation of each consent aspect (supplement a) and asked how important they rated the aspect on a four-point scale from ‘not at all important’ to ‘very important’ and whether they would consent (yes/no). Both questions had an additional ‘I-don’t-know’-response option. Participants could share further thoughts about each aspect in a comments section.

Sociodemographic variables included age, gender, level of education, employment status, and type of insurance.

### Statistical analysis

All analyses were performed with IBM SPSS Statistics version 27 and R version 4.1.2. Demographic characteristics were analyzed using absolute and relative frequencies for categorical data and mean and standard deviations for continuous data. To evaluate the importance ratings, we present absolute and relative frequencies for each aspect.

We utilized nonparametric multiple contrast tests (supplement b) for repeated measures to assess the relative importance of the 14 consent aspects (Rubarth et al. 2022). To rank their relative importance, all aspects were compared to a common reference—the full set of aspects—instead of performing pairwise comparisons. For each item, a relative effect measure ($\:\widehat{{p}_{i})}$ is provided alongside a 95% confidence interval, where $\:\widehat{{p}_{i}}\:$may be interpreted as an estimated tendency towards larger importance of aspect $\:i$ relative to the full set of aspects. Hence, larger relative effect estimates suggest a tendency towards higher importance. Specifically, $\:\widehat{{p}_{i}}$ > $\:\widehat{{p}_{j}}$ indicates that item $\:i$ tends to be ranked as more important than item $\:j$. Note that correction for testing 14 hypotheses is an inherent feature of the procedure, thus corresponding p-values can be interpreted as adjusted for multiple testing. P-values < 0.05 were deemed statistically significant.

We further aimed to assess the relevance of the 14 consent aspects in relation to hypothetical consent to provide insights into consent behavior. We compared the importance ratings between participants who would consent and those who would not, using the nonparametric Brunner-Munzel test, as implemented in the R package *nparcomp* (Version 3.0). The effect estimator is the probability $\:\:\widehat{\boldsymbol{p}}$, representing the likelihood that a random person who consents rates the item as more important than a random person who refuses consent. No correction for multiplicity was applied; the corresponding p-values should be interpreted as exploratory. To descriptively examine the relationship between age and importance ratings, we computed Spearman correlations with 95% confidence intervals.

### Qualitative analysis

A comments section was provided to better capture participants’ reflections and questions while reviewing the consent form. Responses were analyzed according to qualitative content analysis (Mayring 2022). Data were paraphrased, reduced and structured in themes and subthemes. An inductive approach was chosen, which implies that themes were derived from the data.

## Results

### Sample characteristics

A total of 260 individuals with an elevated breast and/or ovarian cancer risk accessed the questionnaire. With 36 who did not provide any information, the final sample consisted of 224 participants. The mean age was 48.4 years (*SD* = 10.9). The sample was predominantly female (97.9%). The education level was high, with 78.1% having obtained the highest school-leaving certificate. Most participants (76.3%) were currently employed, while some were retired (15.1%), or on sick leave (5.4%). The majority (87.7%) was covered by statutory health insurance (supplement c).

### Descriptive information on the subjectively rated importance of consent aspects

With the exception of the items 11 (*digital storage in general hospital information system*,* HIS*) and 13 (*transfer of health data to non-EU country*), all items were rated ‘rather important’ or very ‘important’ by at least three quarters of participants (supplement d). Items 11 and 13 received a notable proportion of rather-not-important-, not-at-all-important-, or I-don’t-know-answers. Yet, those items have a combined importance of just over two-thirds. The items *research purposes* (2), *additional test results* (3), *disclosure of genetic data* (5), *re-evaluations* (9), and *use of test result for relatives* (14) received the highest importance ratings. Over 90% responded with ‘rather important’ or ‘very important’.

### Descriptive information on hypothetical consent

Again, with the exception of the items 11 (yes: 46.3%) and 13 (yes: 51.7%), participants largely stated that they would consent (supplement e). 11/14 items showed consent rates over 80%; specifically, *duration of storage* (1), *research purposes* (2), *re-evaluations* (9), and *use of test result for relatives* (14).

### Comparison of consent aspects

**Fig. 1:**
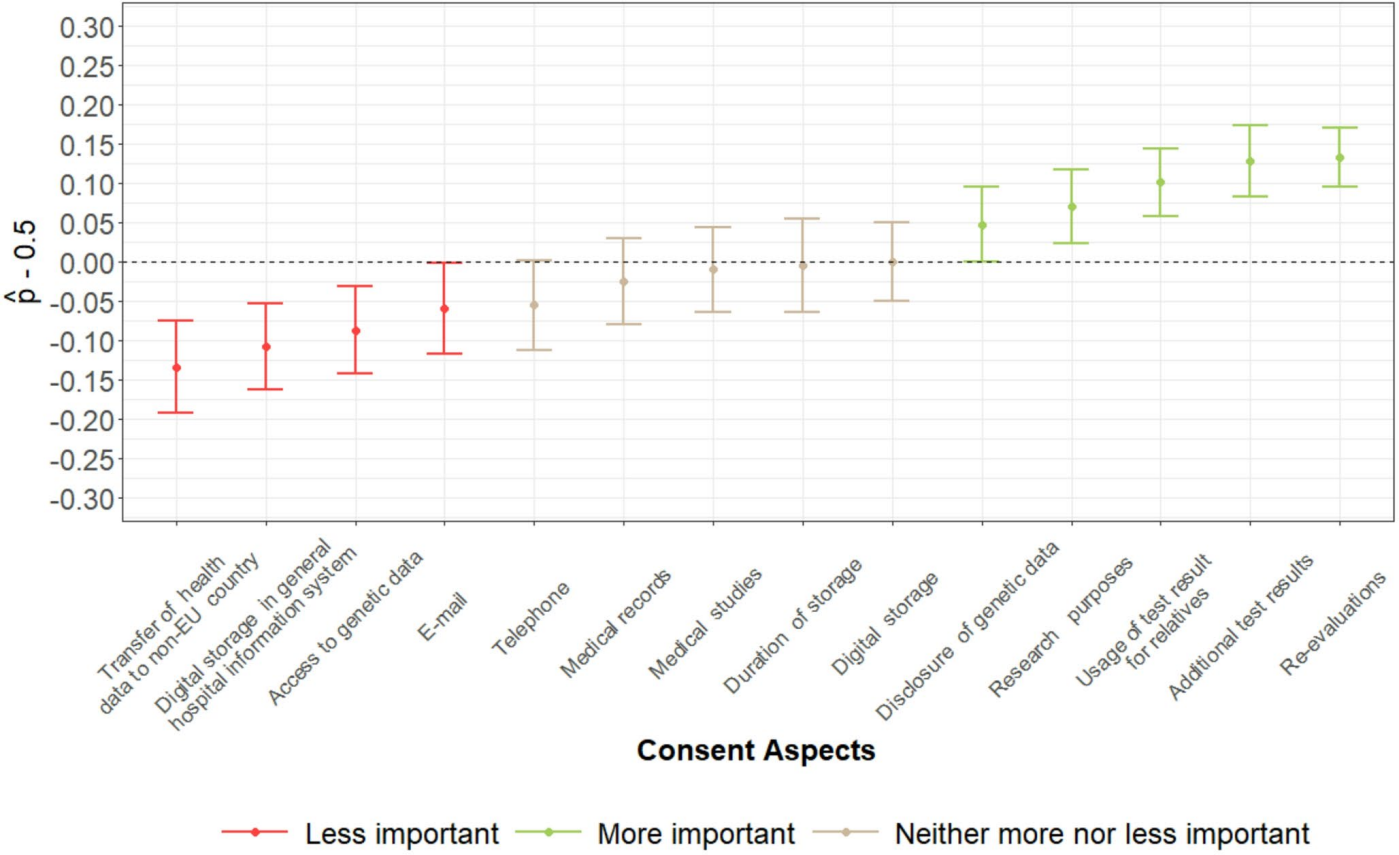
Relative importance of all items in ascending order; items colored red indicate statistically significantly less important items as compared to the mean distribution of all other items, while items colored green indicate statistically significantly more important items

Figure 1 displays the relative differences between the 14 items. Significantly more important were the items *re-evaluations* (9), *additional test results* (3), *use of test result for relatives* (14), *research purposes* (2), and *disclosure of genetic data* (5). Significantly less important were the items *e-mail* (7), *access to genetic data* (4), *digital storage in the general HIS* (11), and *transfer of health data to non-EU country* (13). For detailed statistical analysis see supplement f. There was no significant association between age and the subjectively perceived importance of consent aspects.

### Importance of consent aspects in relation to hypothetical consent

Due to the small number of consent refusals, tests could only be calculated for 11 items (see supplement g). Four items showed statistically significant differences at the 5% level, with large effect sizes ranging from 0.68 to 0.76, indicating a tendency towards higher importance ratings for participants who would hypothetically consent: item 13 (*transfer of health data to non-EU country*; *n* = 141,$\:\widehat{\boldsymbol{p}}$ = 0.68, 95%-CI [0.20, 0.43]), item 6 (*telephone*; *n* = 187, $\:\widehat{\boldsymbol{p}}$ = 0.75, 95%-CI [0.10, 0.40]), item 7 (*e-mail*; *n* = 189,$\:\:\widehat{\boldsymbol{p}}$ = 0.75, 95%-CI [0.05, 0.43]) and item 12 (*medical records*; *n* = 177,$\:\:\widehat{\boldsymbol{p}}$ = 0.76, 95% CI [0.08, 0.40]).

### Patient perspective (qualitative analysis)

Of 224 participants, 25 provided additional thoughts on the consent aspects. Three key themes emerged from the data and are described below, along with a brief summary of patient feedback on response and consent behavior.

#### Theme one: secure handling of genetic data

This theme captured patients’ active engagement with their right to informational self-determination. A strong desire for control over personal data was explicitly expressed by one patient:

*“It is important for me to always retain control over where my data is stored and who has access to my data. In other words*,* I would like to be asked for my consent. (…).”* (patient, age n/a).

Moreover, patients showed a general interest in acquiring genetic knowledge despite considerations and worries about data security. One patient reported the benefits of testing to outweigh their worries:

*“I think it’s a strange feeling that personal health data are stored for so long and ‘sent around everywhere’*,* but it is important and essential for your own medical further care*,* that of your relatives and for research!”* (patient, age 48).

Yet, another patient seemed disillusioned with the idea that health data could really be safe even with adequate measures put in place:

*“Who wants to vouch for the fact that these collected health data are not viewed and*,* or utilized by others*,* you? certainly not.”* (patient, age 63).

Accordingly, several patients concluded that data security is a prerequisite for providing consent. Patients also positively commented on data donation for research purposes, again, under the condition of appropriate security measures:

*“Data security is essential. If it can be guaranteed and misuse is basically ruled out*,* health data should be used extensively for research purposes; in the interests of public health (…).”* (patient, age 59).

#### Theme two: ethical considerations

This theme noted that patients’ experience goes far beyond the testing procedure and acquisition of genetic knowledge. Some patients explicitly expressed a wish for ethical standards to be respected:

*“Everything that was mentioned in the questionnaire was important in compliance with medical confidentiality and consideration for the protection of patient data.”* (patient, age n/a, study ).

Additionally, one patient shared a wish for individual customization of consent choices:

*“I would be happy if I could adapt my decision individually and independently of time in certain situations*.” (patient, age 64).

Another patient stressed that trust in the treating physician or the institution is a decisive factor:

*“(…) Whether I would give my consent also depends on the respective relationship of trust.”* (patient, age 43).

Furthermore, several patients reported the time and place of the disclosure of the test result to be specifically relevant. This was stressed through reports of negative personal experiences and preferences for in-person appointments to discuss the result or other relevant diagnoses:

*“(…) the communication of a positive test result via telephone is difficult because it is quite impersonal and overwhelming. The same applies to email*,* I prefer an emphatic*,* personal conversation.”* (patient, age 40).

#### Theme 3: insurances

Patient responses included an expectation to receive information on insurance matters. Some patients stated that they received insufficient information about insurance matters following genetic testing. One patient particularly reported uncertainty and regret due to this:

*“Can an insurance company obtain genetic data if no questions were asked when the policy was taken out? Unfortunately*,* no information is provided in this regard.”* (patient, age 63).

#### Feedback to questionnaire

Refusal of consent or I-don’t-know-responses were primarily attributed to insufficient information, such as details about personal circumstances of genetic testing.

## Discussion

Our results show that patients at elevated risk for hereditary breast and/or ovarian cancer assign different levels of importance to consent aspects typically presented prior to genetic testing. It should be noted, however, that these are relative differences: in absolute terms, all aspects were considered to be important.

### Consent aspects with direct clinical relevance are most important

The three consent aspects with the highest relative importance all hold clinical relevance for patients and their families: being re-contacted with updated information on a variant of currently uncertain significance, being informed about additional test results, and allowing test results to be used in counseling sessions of relatives^1^. These findings suggest that patients tend to take full advantage of genetic information to address immediate as well as more long-term clinical questions, which was also evident in the qualitative responses. A general desire for detailed genetic information is in line with previous research (Kerševan et al. 2025; Lenk et al. 2019; Wolff et al. 2010). Whereas other studies explored personal motivations for genetic testing or concerns about testing outcomes (Shickh et al. 2023; Zilliacus et al. 2012), our study focused on the relevance of consent aspects.

Interestingly, consent aspects rated as less important were primarily ‘technical’ in nature: preferred mode of contact (e-mail), access to genetic data by other professionals within a clinic, digital storage of genetic data in the general hospital information system (HIS), and transfer of health data for individual risk calculation to a non-EU-country. The latter two aspects also received many I-don’t-know-responses, suggesting that patients are unfamiliar with the meaning of the general HIS or the implications of data transfer outside of the EU. Our assumption, that there is a need for more in-depth explanations, is supported by patient comments indicating that I-don’t-know was sometimes selected due to insufficient available information. With some of the technical items being the main exception, participants were generally willing to provide consent.

Qualitative responses highlighted an awareness of the individual and public benefits of genetic testing, alongside concerns about data misuse and potential insurance restrictions. Participants stressed being informed about disclosure of genetic data and respect of ethical standards to be relevant. They also mentioned the importance of adequate security measures and trust in healthcare providers. Trust has been recognized as a critical factor in patient consent (Hofmann 2023; Roache 2014). For example, studies indicate that breast cancer patients who generally trust their providers take a more active role in their medical care (Arora and Gustafson 2009; Kaiser et al. 2011). In addition, public trust in the healthcare system and government may be relevant and warrants further research. This aligns with ethical discussions emphasizing the importance of institutional trustworthiness and the role of policy and governance structures in supporting informed patients (Sheehan et al. 2019).

### Efforts to improve written consent

It is widely agreed that full understanding is a prerequisite for IC. There remains, however, a gap between this ideal and the realities of clinical practice (Hofmann 2023). Various solutions have been proposed: highlighting key information, improving layout for readability and simplifying language (Coleman et al. 2021; Perrault and Keating 2018). Innovative approaches include comics (Anderson et al. 2016; Brand et al. 2019; Furuno and Sasajima 2015) and digital tools (Gesualdo et al. 2021; Goldschmitt et al. 2025; Miron-Shatz and Yaniv 2022). In the privacy domain, strategies like visual aids (for example privacy icons^2^) could be effective (Efroni et al. 2019; Jurcys et al. 2020). Patients also appreciate written summaries (Köngeter et al. 2022), which can serve as a memory aid (Fischer-Rosinský et al. 2025), and thereby enable them to reflect on their consent choices after counseling. These undoubtedly very important approaches relate to the visual and linguistic representation of consent aspects. Our study provides an empirical basis for the proposal by Ludewigs et al. (2025) to organize consent aspects according to their relevance; ordering items regarding the medical procedure in front of privacy-related items. Our findings highlight the need for further elaboration on specific consent aspects, thereby providing useful insights for improving the communication with counseling physicians (Ludewigs et al. 2025; Springer et al. 2024).

### Suggestions for future research

A subsequent step could involve examining whether a general reduction of consent forms, as suggested by Ben-Shahar and Schneider (2014), is appropriate and legally feasible. It should be assessed whether this goal can be legally achieved by minimizing the technical, particularly privacy-related, part of currently used consent forms by relying on statutory provisions to authorize the respective use of personal data instead of explicit consent by the patient; the GDPR provides for such authorization (Art. 6 I b) – f), Art. 9 II b) – j) GDPR) (Metzger et al. 2023).

### Strengths and limitations

Strengths of our study include the interdisciplinary approach combining empirical legal research, social science, and clinicians with counseling experience. In addition, we applied a novel statistical method that enables the ranking of consent aspects. Limitations include the restriction to the German context, however, the issue of excessively lengthy consent forms is not confined to Germany. Another limitation is the complexity of the hypothetical assessment; still, qualitative responses provided insights into the experience of dealing with the consent form. The majority of participants had already undergone genetic testing and it is likely that highly motivated individuals participated, both of which implies a potential bias. increase the complexityA general limitation of anonymous online studies is that it is not possible to make a valid statement about the response rate. However, given that the distribution of sociodemographic variables is similar to that reported in previous studies with the same patient population (Kendel et al. 2022; Feufel et al. 2024), the sample appears to be broadly representative. Finally, future studies could benefit from including data on ethnicity and cultural background to adequately support diverse patients.

## Conclusion

Patients do not consider all aspects of consent to be equally important. In the context of genetic testing, they seem to prioritize aspects offering direct clinical value, while placing relatively less importance on more technical aspects of data use. Apart from the ranking, the qualitative results reveal a general interest in acquiring genetic knowledge, and emphasize the importance of trust and adequate security measures. The complexity of consent forms may be reduced by moving the most important aspects to more prominent positions and highlighting them accordingly. Future efforts should empirically investigate the phenomenon of ‘consent fatigue’ and examine how reduced consent forms can support decision-making and promote patient autonomy. Finally, the empirical basis as well as the lived experiences of patients with a family history of cancer may inform policy and regulatory reform.

## Supplementary Information

Below is the link to the electronic supplementary material.

Supplementary Material 1

## Data Availability

The data that support the findings of this study are available upon request from the corresponding author.

## Footnotes

1 At the HBOC-center at Charité - University Medical Center Berlin this consent item is no longer provided for data protection reasons. As noted in the Genetic Diagnostics Act (§ 11 (1)), testing is a very private matter which first and foremost concerns the patient and the counselling physician. The patient, of course, has the right to disclose the result to others, for instance their relatives.

2 Cf. Definition of Berkman Klein Center, online available at: https://cyber.harvard.edu/i4bi/Privacy_Icons.
