# Supplementary material for: Not all information is equally important: informed consent to genetic testing for hereditary cancer
Source: J Cancer Res Clin Oncol. 2026 Jan 27;152(2):44. doi: 10.1007/s00432-026-06422-y (PMC12847596; doi:10.1007/s00432-026-06422-y)
Supplement: Supplementary file 1 — Supplementary Material 1 [file 432_2026_6422_MOESM1_ESM.docx]

**Supplementary material**

1. **Overview of all 14 consent aspects which were presented as one item each on the questionnaire provided for participants**

| Full item description given to participants | Abbreviation |
| --- | --- |
| 1) Duration of storage of genetic test result  *It is a legal requirement that the test result is stored for 10 years. If the storage period is longer, the result can also be used e.g. for the counseling or treatment of biological relatives, if you also consent to this purpose.* | Duration of storage |
| 2) Use of remaining test material for research purposes  *The genetic test is usually a blood test. After the test, some of the test material remains unused. This material can e.g. be used for research.* | Research purposes |
| 3) Notification of additional test results  *Additional test results are results that your physician did not aim to investigate, but which were discovered during the genetic test. An additional test result can e.g. be an increased risk for another type of cancer.* | Additional test results |
| 4) Access to genetic data by employees within a medical practice or center  *Besides the treating physician, there are other healthcare professionals who require access benefit from having access to genetic data in order to be involved in your care.* | Access to genetic data |
| 5) Disclosure of test result to other treating physicians  *The result of the genetic test may be disclosed to other physicians, such as the General Practitioner (GP).* | Disclosure of genetic data |
| 6) Contact by telephone  *Your contact details will be requested so that you can be contacted by telephone, e.g. if you have any queries about the genetic test, for notification of the result or to make an appointment.* | Telephone |
| 7) Contact by e-mail  *Your contact details will be requested so that you can be contacted by e-mail, e.g. if you have any queries about the genetic test, for notification of the result or to make an appointment.* | E-mail |
| 8) Contact for participation in medical follow-up studies  *Even if a long time has passed since your appointment for genetic counseling, it may become apparent that you are eligible to take part in a medical study. With your consent, you can be contacted for participation.* | Medical studies |
| 9) Contact for re-evaluation of test result  *In certain cases, new information on genetic findings may emerge months or years after your genetic counselling appointment. With your consent, you can be informed about these findings.* | Re-evaluations |
| 10) Digital storage of genetic data  *As part of the consent to genetic testing, you have to decide whether your genetic data may also be stored digitally.* | Digital storage |
| 11) Digital storage of genetic data in the general hospital information system (HIS)  *Due to the sensitive nature of genetic data, it is usually stored on a secure server which only certain members of medical staff have access to. With your consent, it would also be possible to store genetic data in the general hospital information system so that other physicians providing treatment can also access it.* | Digital storage in general hospital information system |
| 12) Including genetic data in medical records  *Medical records are written to inform other treating physicians, such as your gynecologist or GP, but not health insurance companies.* | Medical records |
| 13) Transfer of health data to a university in a non-EU country to calculate the individual risk of disease  *The individualized cancer risk can be calculated using a computer program. For the calculation, information on your family history regarding cancer, your year of birth and the genetic test result are required. This information can then be transferred to the University of Cambridge (United Kingdom) for risk calculation.* | Transfer of health data to non-EU country |
| 14) Use of test result for counseling of biological relatives  *To ensure that the genetic test result can also be used for counseling of biological relatives, you must release the treating physician from their duty of confidentiality.* | Use of test result for relatives |

1. **Mathematical appendix**

Multiple contrast tests were calculated in R version 4.1.2 to rank the importance items. A brief overview of the mathematical principles used, tailored to our application, is given here. For a more detailed and general discussion we refer to the work by Rubarth, Pauly and Konietschke (2022).

Since $14$ importance items were asked per person and the multiple contrast tests are based on $n= 150$ participants, our data can be described by $150$ independent and identically distributed $14$-dimensional random vectors

$X_{k}=\left( \left( \lambda_{1k},X_{1k} \right),\ldots, \left( \lambda_{14k},X_{14k} \right) \right)', k=1,\ldots, 150$, with

$$\lambda_{ik}=\left\{ \begin{aligned} 1, X_{ik} is observed \\ 0, X_{ik} is missing, \end{aligned} \right.$$

and the marginal distribution $X_{ik}\sim F_{i}\left( x \right), i=1,\ldots, 14$, where $X_{ik}$ represents the importance rating of subject $k$to item $i$ . To compare the importance of the 14 items, we use unweighted relative effects

$$p_{i}=P\left( Z<X_{i1} \right)+\frac{1}{2}\cdot P\left( Z=X_{i1} \right), i=1,\ldots, 14,$$

where $Z\sim G(x)$ represents a random variable following the unweighted mean distribution of all $F_{i}$ Effects are computed relative to the mean distribution rather than across all pairwise item combinations, as pairwise effects are non-transitive and may yield paradoxical outcomes. In our interpretation, item $i$ tends to be more important than item $j$ if $p_{i}>p_{j}$ and neither tends to be less or more important if $p_{i}=p_{j}$. The information of the comparisons of all individual items to the mean distribution is encoded in a Grand Mean-type contrast matrix $C\in\mathbb{R}^{14\times14}$, where $c_{l}^{'}$ denotes its $l$-th row. The contrast matrix was generated in R using the multcomp package (version 1.4-25; (Hothorn et al. 2008)). Replacing the distribution functions $F_{i}(x)$ and $G(x)$ by their empirical counterparts leads to a point estimator $\hat{p}$, which is used to rank all items according to their relative importance

Using an estimator for the covariance matrix $\hat{V}_{n}\in\mathbb{R}^{14\times14}, n=150$, test statistic for each contrast can be written as

$$T_{l}=\sqrt{n} \cdot\frac{c_{l}^{'}\left( \hat{p}-p \right)}{\sqrt{c_{l}^{'}\hat{V}_{n}c_{l}}}, l=1,\ldots, 14 \mathrm{and} n=150.$$

Finally, the distribution of the vector of test statistics $\left( T_{1},\ldots,T_{14} \right)$ can be approximated by a multivariate central t-Distribution with expectation $0$, correlation matrix $\boldsymbol{R}$**,** where $\boldsymbol{R}$ can be estimated from the data, and $n-1$ degrees of freedom. Based on this multivariate t-Distribution, $p$-value can be computed. The confidence intervals are obtained by

$${Cl}_{l}=\left[ c_{l}^{'}\hat{p} \mp\frac{t_{1-\alpha,\boldsymbol{R,n-1}}}{\sqrt{n}}\sqrt{c_{l}^{'}\hat{V}_{n}c_{l}} \right], l=1,\ldots, 14 \mathrm{and} n=150,$$

where $t_{1-\alpha,\boldsymbol{R,n-1}}$ represents the $(1-\alpha)$-equicoordinate quantile from the multivariate t-Distribution above.

1. **Sociodemographic information** provided by *n*= 194 without missing values

| **Variable** | |  |
| --- | --- | --- |
| **Age** (years), Mean (*SD*) | | 48.4 (10.9) |
| **Gender**, *n* (%) | |  |
|  | Female | 182 (97.9) |
|  | Male | 2 (1.1) |
|  | Diverse | 2 (1.1) |
| **Relationship status**, *n* (%) | |  |
|  | In a relationship | 152 (82.6) |
|  | Not in a relationship | 32 (17.4) |
| **Education**, *n* (%) | |  |
|  | University entrance qualification (*Fachhochschulreife/Abitur)* | 146 (78.1) |
|  | Intermediate school-leaving certificate *(Realschule/Mittlere Reife)* | 40 (21.4) |
|  | Leaving certificate *(Hauptschule/Volksschule)* | 1 (0.5) |
|  | No qualification | 0 (0.0) |
| **Occupation**, *n* (%) | |  |
|  | Employed | 142 (76.3) |
|  | In education | 3 (1.6) |
|  | Unemployed | 3 (1.6) |
|  | Currently unable to work | 10 (5.4) |
|  | Retired | 28 (15.1) |
| **Type of health insurance**, *n* (%) | |  |
|  | Statutory | 164 (87.7) |
|  | Private | 23 (12.3) |

1. **Subjectively rated importance of all 14 consent aspects**

| Item | **Subjectively reported importance, *n* (%)** | | | | |
| --- | --- | --- | --- | --- | --- |
|  | **Not at all important** | **Rather not important** | **Rather important** | **Very important** | **I don’t know** |
| 1) Duration of storage of genetic test result (*n*=223) | 4 (1.79) | 24 (10.76) | 67 (30.04) | 125 (56.05) | 3 (1.35) |
| 2) Use of remaining test material for research purposes (*n*=217) | 1 (0.46) | 11 (5.07) | 50 (23.04) | 154 (70.97) | 1 (0.46) |
| 3) Notification of additional test results (*n*=214) | 0 (0.00) | 2 (0.93) | 33 (15.42) | 172 (80.37) | 7 (3.27) |
| 4) Access to genetic data by employees within a medical practice or center (*n*=209) | 5 (2.39) | 23 (11.00) | 84 (40.19) | 81 (38.76) | 16 (7.66) |
| 5) Disclosure of test result to other treating physicians (*n*=208) | 1 (0.48) | 9 (4.33) | 69 (33.17) | 126 (60.58) | 3 (1.44) |
| 6) Contact by telephone (*n*=207) | 6 (2.90) | 29 (14.01) | 74 (35.75) | 89 (43.00) | 9 (4.35) |
| 7) Contact by  e-mail (*n*=205) | 4 (1.95) | 19 (9.27) | 87 (42.44) | 88 (42.93) | 7 (3.41) |
| 8) Contact for participation in medical follow-up studies (*n*=205) | 1 (0.49) | 14 (6.83) | 81 (39.51) | 102 (49.76) | 7 (3.41) |
| 9) Contact for re-evaluation of test result (*n*=205) | 0 (0.00) | 0 (0.00) | 41 (20.00) | 161(78.54) | 3 (1.46) |
| 10) Digital storage of genetic data (*n*=206) | 2 (0.97) | 14 (6.80) | 71 (34.47) | 105 (50.97) | 14 (6.80) |
| 11) Digital storage of genetic data in the general hospital information system (*n*=205) | 9 (4.39) | 29 (14.15) | 74 (36.10) | 68 (33.17) | 25 (12.20) |
| 12) Including genetic data in medical records (*n*=205) | 3 (1.46) | 14 (6.83) | 76 (37.07) | 95 (46.34) | 17 (8.29) |
| 13) Transfer of health data to a university in a non-EU country to calculate the individual risk of disease (*n*=203) | 9 (4.43) | 29 (14.29) | 75 (36.95) | 60 (29.56) | 30 (14.78) |
| 14) Use of test result for counseling of biological relatives (*n*=203) | 0 (0.00) | 1 (0.49) | 42 (20.69) | 155 (76.35) | 1. (2.46) |

*Note*. Participants who did not rate the importance of any item were excluded from the data set. Their data was assumed to be missing completely at random. The information in the table refers to the data set without taking missing values into account. Therefore, the total n for each item respectively is reported.

1. **Hypothetical consent to each of the 14 consent aspects**

| Item | **consent, *n* (%)** | | |
| --- | --- | --- | --- |
|  | **yes** | **no** | **I don’t know** |
| 1) Duration of storage of genetic test result (*n*=223) | 206 (92.38) | 7 (3.14) | 10 (4.48) |
| 2) Use of remaining test material for research purposes (*n*=217) | 199 (91.71) | 9 (4.15) | 9 (4.15) |
| 3) Notification of additional test results (*n*=214) | 192 (89.72) | 1 (0.47) | 21 (9.81) |
| 4) Access to genetic data by employees within a medical practice or center (*n*=209) | 148 (70.81) | 28 (13.40) | 33 (15.79) |
| 5) Disclosure of test result to other treating physicians (*n*=208) | 179 (86.06) | 15 (7.21) | 14 (6.73) |
| 6) Contact by telephone (*n*=207) | 174 (84.06) | 18 (8.70) | 15 (7.25) |
| 7) Contact by e-mail (*n*=205) | 180 (87.80) | 14 (6.83) | 11 (5.37) |
| 8) Contact for participation in medical follow-up studies (*n*=205) | 183 (89.27) | 2 (0.98) | 20 (9.76) |
| 9) Contact for re-evaluation of test result (*n*=205) | 202 (98.54) | 0 (0.00) | 3 (1.46) |
| 10) Digital storage of genetic data (*n*=206) | 160 (77.67) | 13 (6.31) | 33 (16.02) |
| 11) Digital storage of genetic data in the general hospital information system (*n*=205) | 95 (46.34) | 61 (29.76) | 49 (23.90) |
| 12) Including genetic data in medical records (*n*=205) | 167 (81.46) | 14 (6.83) | 24 (11.71) |
| 13) Transfer of health data to a university in a non-EU country to calculate the individual risk of disease (*n*=203) | 105 (51.72) | 44 (21.67) | 54 (26.60) |
| 14) Use of test result for counseling of biological relatives (*n*=203) | 194 (95.57) | 7 (3.45) | 1. (0.99) |

*Note.* The information refers to the data set without taking missing values into account. Therefore, the total n for each item respectively is reported.

1. **Consent aspects ordered according importance**

| **Item** | $\hat{\boldsymbol{p}}$ | $\hat{\boldsymbol{p}}\boldsymbol{-0.5}$ | **95%-CI** | **p-value** |
| --- | --- | --- | --- | --- |
| Re-evaluation (9) | 0.6330 | 0.1330 | [0.0954, 0.1705] | ***<* 0*.*001** |
| Additional test results (3) | 0.6286 | 0.1286 | [0.0830, 0.1742] | ***<* 0*.*001** |
| Use of test result for relatives (14) | 0.6012 | 0.1012 | [0.0584, 0.1440] | ***<* 0*.*001** |
| Research purposes (2) | 0.5705 | 0.0705 | [0.0237, 0.1173] | ***<* 0*.*001** |
| Disclosure of genetic data (5) | 0.5479 | 0.0479 | [0.0008, 0.0950] | **0.0437** |
| Digital storage (10) | 0.5002 | 0.0002 | [-0.0490, 0.0494] | 1.000 |
| Duration of storage (1) | 0.4953 | -0.0047 | [-0.0637, 0.0542] | 1.000 |
| Medical studies (8) | 0.4901 | -0.0099 | [-0.0644, 0.0446] | 1.000 |
| Medical records (12) | 0.4753 | -0.0247 | [-0.0795, 0.0301] | 0.923 |
| Telephone (6) | 0.4453 | -0.0547 | [-0.1116, 0.0021] | 0.069 |
| E-Mail (7) | 0.4409 | -0.0591 | [-0.1169, -0.0012] | **0.042** |
| Access to genetic data (4) | 0.4131 | -0.0869 | [-0.1423, -0.0315] | ***<* 0*.*001** |
| Digital storage in general hospital information system (11) | 0.3920 | -0.1080 | [-0.1628, -0.0532] | ***<* 0*.*001** |
| Transfer of health data to non-EU country (13) | 0.3666 | -0.1334 | [-0.1918, -0.0751] | ***<* 0*.*001** |

*Note.* N = 224 individuals accessed the questionnaire. n = 74 individuals with at least one ‘I don’t know’ answer were excluded. Thus, the statistical analysis was based on n = 150 individuals, including those with < 14 missing values, i. e. incomplete cases were also included. All 14 items are ordered by estimated importance in a descending order. Items colored green indicate statistically significantly more important items as compared to the mean distribution of all other items, while items colored red indicate statistically significantly less important items. The table provides an effect estimator, relative effect measure alongside a 95% confidence interval and p-value for each item.

1. **Subjectively rated importance of the 14 consent aspects in relation to the hypothetical consent**

| **Item** | ***N*** | **consent** | | $\hat{\boldsymbol{p}}$ | $\hat{\boldsymbol{SE}}$ | **95%-CI** | **p-value** |
| --- | --- | --- | --- | --- | --- | --- | --- |
|  |  | **yes** | **no** |  |  |  |  |
| Duration of storage (1) | 211 | 204 | 7 | 0.4426 | 0.1028 | [0.3091, 0.8057] | 0.596 |
| Research purposes (2) | 208 | 199 | 9 | 0.5343 | 0.0886 | [0.2635, 0.6679] | 0.708 |
| Access to genetic data (4) | 166 | 139 | 27 | 0.5472 | 0.0711 | [0.3073, 0.5984] | 0.513 |
| Disclosure of genetic data (5) | 194 | 179 | 15 | 0.5622 | 0.0823 | [0.2622, 0.6134] | 0.462 |
| Telephone (6) | 187 | 169 | 18 | 0.7472 | 0.0709 | [0.1040, 0.4016] | **0.003** |
| E-mail (7) | 189 | 176 | 13 | 0.7579 | 0.0872 | [0.0528, 0.4314] | **0.012** |
| Digital storage (10) | 168 | 156 | 12 | 0.4249 | 0.0847 | [0.3904, 0.75979 | 0.393 |
| Digital storage in general hospital information system (11) | 153 | 95 | 58 | 0.5901 | 0.0503 | [0.3097, 0.5100] | 0.077 |
| Medical records (12) | 177 | 163 | 14 | 0.7603 | 0.0761 | [0.0763, 0.4031] | **0.004** |
| Transfer of health data to non-EU country (13) | 141 | 101 | 40 | 0.6821 | 0.0562 | [0.2047, 0.4312] | **0.002** |
| Use of test result for relatives (14) | 197 | 192 | 5 | 0.5922 | 0.1228 | [0.0709, 0.7447] | 0.493 |
